# Efficacy and Safety of Medical Marijuana in Migraine Headache: A Systematic Review

**DOI:** 10.7759/cureus.32622

**Published:** 2022-12-17

**Authors:** Mingma L Sherpa, Nilasma Shrestha, Blessing T Ojinna, Niriksha Ravi, Vivig Shantha Kumar, Silpa Choday, Anusha Parisapogu, Hadrian Hoang-Vu Tran, Anil KC, Abeer O Elshaikh

**Affiliations:** 1 Internal Medicine and Neurology, California Institute of Behavioral Neurosciences & Psychology, Fairfield, USA; 2 Pathology and Internal Medicine, California Institute of Behavioral Neurosciences & Psychology, Fairfield, USA; 3 Internal Medicine, California Institute of Behavioral Neurosciences & Psychology, Fairfield, USA; 4 Infectious Disease, California Institute of Behavioral Neurosciences & Psychology, Fairfield, USA; 5 Internal Medicine/Family Medicine, California Institute of Behavioral Neurosciences & Psychology, Fairfield, USA

**Keywords:** headaches, migraine, cannabinoids, cannabis, marijuana

## Abstract

Medical marijuana treatment for migraine is becoming more common, although the legality and societal acceptance of marijuana for medical purposes in the United States have been challenged by the stigma attached to it as a recreational drug. These substances function to reduce nociception and decrease the frequency of migraine by having an impact on the endocannabinoid system. Our study reviewed the clinical response, dosing, and side effects of marijuana in migraine management. Using Preferred Reporting Items for Systematic Reviews and Meta-Analyses (PRISMA) guidelines, we conducted a literature search in PubMed, Google Scholar, and Science Direct, and nine studies were included in the systematic review. The studies demonstrated that medical marijuana has a significant clinical response by reducing the length and frequency of migraines. No severe adverse effects were noted. Due to its effectiveness and convenience, medical marijuana therapy may be helpful for patients suffering from migraines. However, additional clinical trials and observational studies with longer follow-ups are required to study the efficacy and safety of the drug.

## Introduction and background

According to the International Classification of Headache Disorders third edition (ICHD-3), migraine is defined as a recurring disorder with at least five headaches lasting four to seventy-two hours; at least two of the following characteristics, such as unilateral location, pulsating quality, moderate to severe pain intensity, aggravation by causing avoidance of regular physical activity, nausea and/or vomiting, or photophobia and phonophobia, and is not better explained by another ICHD-3 diagnosis [1]. It is one of the leading causes of disability and suffering around the world [2]. In 2013, migraine was classified as the sixth leading cause of years lost due to disability worldwide [2]. It can be episodic or chronic and can be with or without aura [3]. The causes of migraine headaches have been well-researched over many decades but are still up for debate due to their high prevalence and disruptive character [4]. The onset of migraine attacks has been connected to both environmental and hormonal stimuli, which result in pathophysiological alterations caused by sterile neurogenic inflammation in the meninges and activation of trigeminal sensory nerves [4]. Triptans, non-steroidal anti-inflammatory drugs (NSAIDs), paracetamol, ergots, opioids, and antiemetics have all been used in the past to treat abortive migraines [5]. Antidepressants, anticonvulsants, beta-blockers, and, more recently, anti-calcitonin gene-related peptide (CGRP) medicines are among the preventive medications available [5]. But many anti-migraine medications have side effects, posing a difficulty that has led to the halt of research and development of prospective anti-migraine drugs [4]. As a result, the current situation necessitates more investigation, particularly for individuals who do not benefit from or tolerate regularly recommended drugs [6]. Evidence-based guidelines from the United States, Canada, and Europe also offer consistent advice, such as lifestyle changes, trigger avoidance, and coping techniques [7].

Cannabis has been used to cure headaches for hundreds to thousands of years, and medical marijuana users frequently use it today [8]. It has been used for medical purposes since ancient times and has also been recommended by many physicians for many illnesses, especially in the symptomatic and prophylactic treatment of headaches and migraines [9]. Delta-nine-tetrahydrocannabinol (THC) and Cannabidiol (CBD) are the two main bioactive cannabinoids [1].

**Figure 1:**
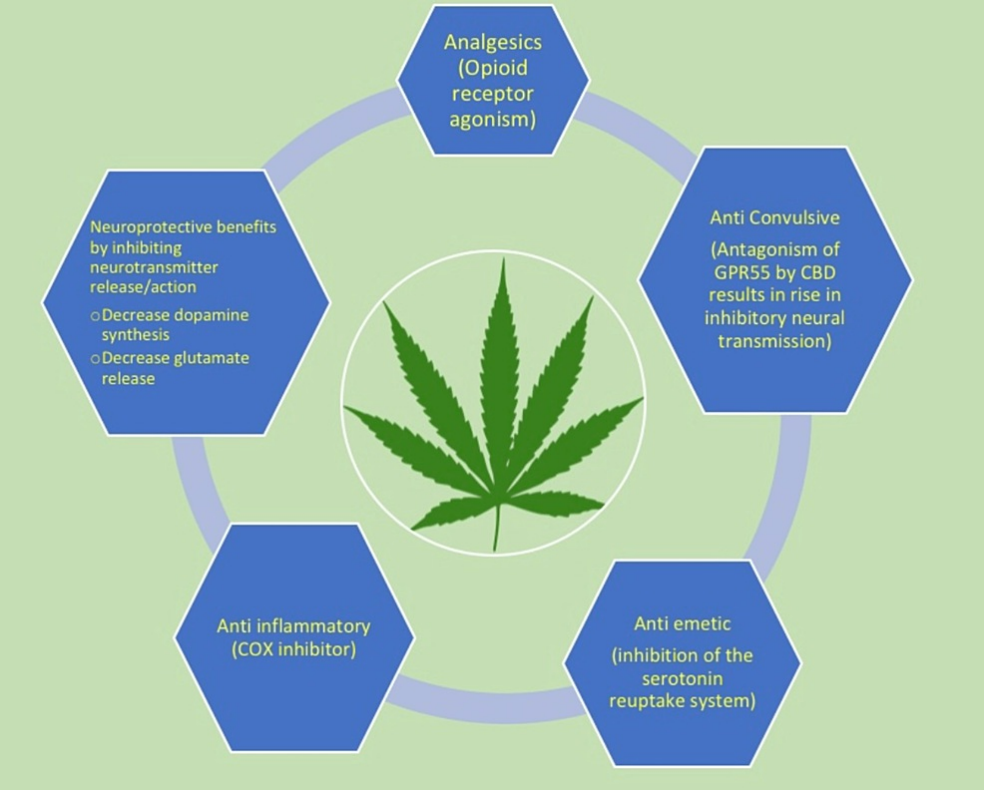
Function of medical marijuana in migraine patients. GPR55: G-protein-coupled receptor 55; CBD: Cannabidiol; COX: Cyclooxygenase

THC predominantly acts as an agonist on cannabinoid receptor one (CB1R), which is shared by the endocannabinoid N-arachidonylethanolamine (AEA, anandamide) [1]. Many areas involved in migraine pathogenesis, such as thalamic pain relays, basal ganglia activity, and cerebellar regulation, are controlled by activity following an AEA-CB1R interaction [1]. To put it simply, THC uses endogenous G-protein-coupled receptor (GPCR) signaling to repair migraine-related pathways [1]. THC can also act as a dopamine antagonist when given in larger doses [10]. Dopamine antagonists have been proven to be particularly helpful at treating migraine attacks, and most antipsychotic drugs and antiemetics are used for headache treatment because of their antidopaminergic effects [10]. Cannabis also shares a similar mechanism of action with headache and migraine drugs in that they both lower plasma glutamate levels [10]. Because magnesium levels are frequently low during headaches and migraines, one explanation for these connections could be glutamate's function in the brain's absorption of magnesium [10]. This may help to explain how N-methyl-D-aspartate (NMDA) antagonists and magnesium supplements can effectively prevent and treat headache- and migraine-related suffering [10]. Compared to THC, CBD's effects on the body are more complex [1]. THC's effects are primarily mediated through CB1R, but CBD's affinity for cannabinoid receptors in the active site is negligible [1]. Both metabotropic and ionotropic actions are a part of how CBD works [1]. By inhibiting G-protein-coupled receptor 55 (GPR55), CBD's metabotropic effects include balancing excitatory brain signaling [1]. The ionotropic effects of CBD include a decrease in sodium current through the stabilization of lipid membranes connected to voltage-gated sodium channels (Na V) [1]. CBD's antagonistic effects on GPR55 cause a rise in inhibitory neural transmission, which may help migraine and seizure patients by calming their brains [1,10]. Figure 1 given below illustrates the function of medical marijuana in migraine patients.

The endocannabinoid system (ECS) is a broad signaling network that is found in almost all cell types and is essential for preserving bodily homeostasis [4]. The central and peripheral nervous systems' stress-responsive regions have an active ECS that works to lessen pain and prevent neurodegeneration and inflammatory damage [4]. There is evidence that endocannabinoids cause plastic changes in numerous brain regions that control pain perception in their short-term effects [4]. All of these pathways are connected to migraine pathology, either directly or indirectly [4]. According to the Clinical Endocannabinoid Deficiency (CED) theory, endogenous cannabinoids, particularly in chronic migraine, may be involved in migraine pathophysiology [1]. The ECS is primarily relayed by two receptors: CB1R, which is one of the most abundant GPCRs in the brain, and cannabinoid receptor two (CB2R), which is functionally linked to CB1R but is found largely in peripheral tissues [4]. Both CB1R and CB2R are activated by endogenous cannabinoids two-arachidonoylglycerol (2-AG) and AEA, but they also react when phytocannabinoids are bound [4]. THC is therefore assumed to primarily function by powerfully activating CB1R and CB2R [4]. There is also evidence that suggests people with chronic migraines have much lower levels of AEA in their cerebral fluid than people without migraines [1]. In the population with chronic migraines, AEA reuptake and metabolism enzyme levels have also been found to be significantly decreased [1].

The goal of this systematic review is to study the efficacy of marijuana to treat migraine headaches. It focuses on how medical marijuana can lessen migraine length and frequency, and also shows various forms and optimal doses used for therapeutic success.

## Review

Method

Study Protocol

We implemented Preferred Reporting Items for Systematic Review and Meta-Analysis (PRISMA) 2020 guidelines in our study process [11].

Source of Data Collection

PubMed, Google Scholar, and Science Direct were searched for relevance. Databases were searched using the keywords and medical subject heading (MeSH) keywords. MeSH strategy was used in PubMed, while keywords were used mainly in other databases.

The keywords used in our search were under two search themes and combined using the Boolean operates ‘AND’ and ‘OR’. For the theme ‘Marijuana’, we used keywords like marijuana, cannabis, cannabinoids, and tetrahydrocannabinol. For the theme ‘Migraine’, we used keywords like migraine and acute and chronic migraine.

The databases used for the search are mentioned in Table 1.

**Table 1:** Databases and search results.

Databases	Search Results	
	Initial Results	Timeframe 2016 to 2022
PubMed	111	68
Google Scholar	1820	1010
Science Direct	1569	568

Inclusion and Exclusion Criteria

We only included the articles published in the English language, which were human studies and clinical trials. We selected articles published from 2016 to 2022. The inclusion criteria were patients diagnosed with migraine, patients with migraine who received marijuana, adult patients between 18 and 60 years old, and both male and female patients. Exclusion criteria were studies on pediatric patients below age 18 and animal studies.

Data Extraction and Quality Assessment

The articles were retrieved first based on the title and abstract related. Then two authors (Mingma and Nilasma) assessed the data independently and screened all the identified studies. Nine papers were selected in the end after the screening process and quality appraisal. For the quality assessment, we used the assessment of multiple systematic reviews (AMSTAR) for systematic review and meta-analysis, the appraisal tool for cross-sectional studies (AXIS) for cross-sectional studies, the Newcastle-Ottawa scale for observational studies, and the scale for the quality assessment of narrative review articles (SANRA) for review articles. Only those screened articles that satisfied >70% of the checklist quality parameters were included in our study.

Results

**Figure 2:**
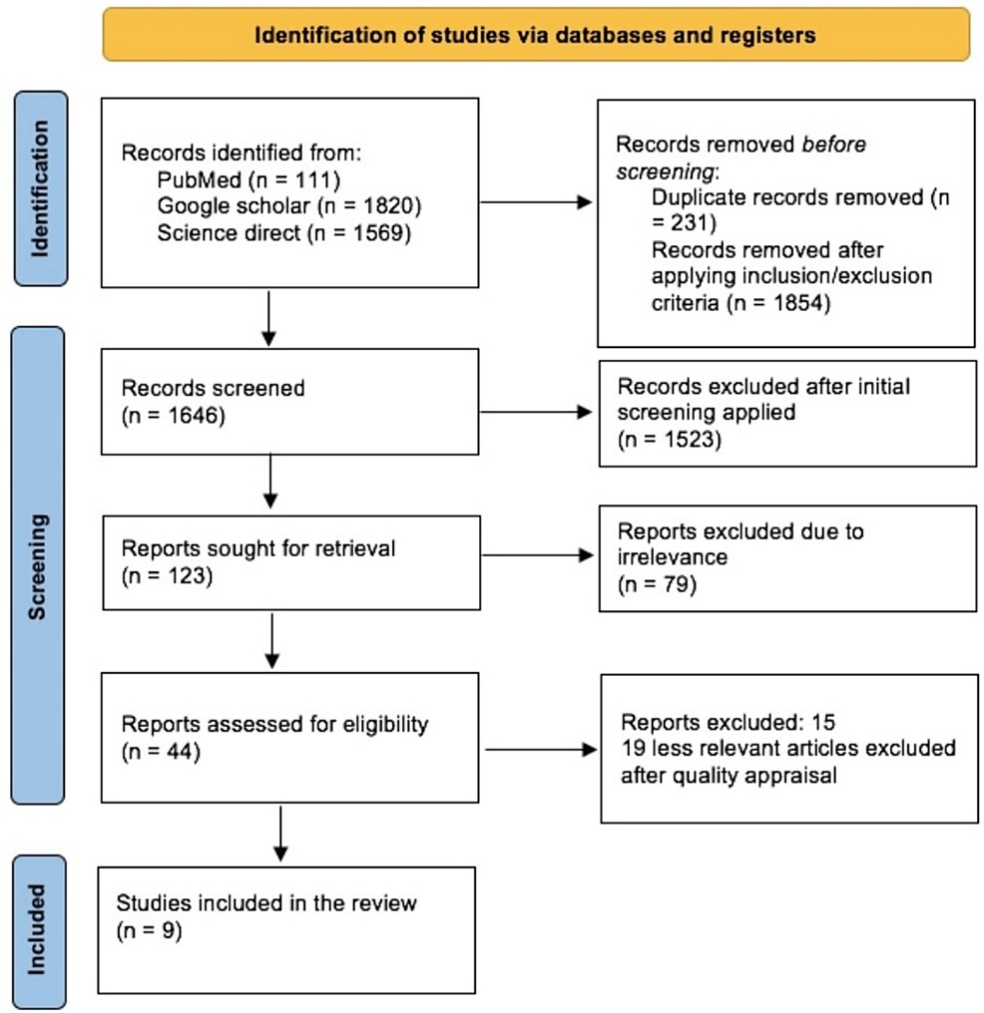
PRISMA flow diagram (2020) showing the search results and selection process. PRISMA: Preferred Reporting Items for Systematic Reviews and Meta-Analysis

A total of 3500 articles were identified in our initial search of the PubMed, Google Scholar, and Science Direct databases. All database references were formatted using Endnote (Clavirate Analytics, Philadelphia, USA), and 231 duplicates were removed using Rayyan software (Rayyan Systems Inc., Cambridge, Massachusetts). Out of these articles, 1854 were discarded either due to duplication or not being directly relevant to our research focus, leaving us with 1646 articles to analyze. After the detailed assessment, and applying inclusion/exclusion criteria, we ended up with 123 articles and excluded 1531 articles. In the end, only nine articles were included, and these articles were checked for quality based on their study characteristics [1,5,8,10,12-16]. A complete PRISMA flow diagram is given below in Figure 2.

Discussion

Efficacy of Medical Marijuana in the Treatment of Migraine Headache

In 2016, Rhyne et al. conducted a retrospective study using reviews of medical records, with the primary objective of analyzing the frequency of headaches caused by medical marijuana and the secondary objective of concentrating on the kind, dosage, prior usage of migraine medications, and patient-reported data [12]. A retrospective chart assessment of 121 patients in Colorado using medical cannabis for migraine disease found that 103 patients (85.1%) experienced a reduction in migraine frequency over an average of 21.8 months [12]. They also found that migraine sufferers who inhaled medical cannabis experienced a significant decrease in migraine frequency [12].

In 2019, in their survey study using a Canadian data application (Strainprint, Strainprint Technologies Ltd., Ontario, CA), Cuttler et al. centered on the use of inhaled cannabis and how it affected the severity and frequency of migraines as well as the variables influencing the dosage utilized [8]. According to survey findings, headaches were reduced by 47.3% and migraines by 49.6% [8]. Females (88.6%) compared to males (87.3%) reported a more favorable reduction in headaches, while a higher percentage of men (90.9%) compared to women (89.1%) reported a more favorable reduction in migraines [8]. A total of 88.1% of patients reported a reduction in migraine severity with inhaled cannabis over the course of 16 months, as evidenced by 7441. Four hours of sessions between 653 patients [8]. A higher pre-inhalation severity rating corresponded to greater reductions in the sample's mean migraine severity rating, which decreased by 49.6% (pre-6.65, post-3.30) [8]. The average THC concentration was 14.9%, ranging from 0 to 77%, and the average CBD concentration was 2.58%, ranging from 0 to 50.7% [8]. These large variations make it clear that neither the THC nor CBD concentration had any influence on reducing severity [8]. Regardless of dosage, cannabis preparation, flower, or concentration, severity was also decreased [8]. It also looked into how tolerance changes with continued cannabis consumption [8]. This study's weaknesses included the absence of a control group and biased sampling [8].

Similarly, in a study conducted by Stith et al. in 2019, they found that discomfort from migraines and headaches can be efficiently treated with dried cannabis flowers [10]. The researchers used the Releaf application (Releaf, Hyattsville, US) to record real-time information about their cannabis usage, including product features and symptom intensity levels before and after self-administration from 699 participants between 2016 and 2019 [10]. A total of 1910 sessions of attempts to treat headache (1328 sessions) or migraine-related pain (582 sessions) were included in the data [10]. Ninety-four percent of participants in 1,910 sessions reported symptom relief within a two-hour observation window [10]. Users' gender, age, whether they experienced headaches or migraines, and cannabis strain variances all had an impact on alleviation trends [10].

Gibson et al. did a cross-sectional study in 2020, where participants responded to an online survey to evaluate their cannabis usage patterns, migraine experiences, and self-reported relief from cannabis and non-cannabis treatments [13]. The survey shed light on the frequency of cannabis use for migraine relief in a sample of cannabis users and implies that these migraineurs benefit greatly from cannabis use [13]. A total of 161 individuals (27.3%) mentioned having migraines [13]. A total of 76.4% of migraine sufferers (N = 123) supported utilizing cannabis as a migraine treatment [13]. When it comes to treating their migraines, 69.9% (N = 86) of those who use cannabis for this purpose also support utilizing triptans and other non-cannabis medications [13]. Despite having identical subjective health (p = .17), migraineurs who supported using cannabis as a migraine treatment reported having more severe migraines than those who did not (p = .02) [13]. Even after controlling for migraine severity, cannabis users reported considerably higher migraine relief than users of non-cannabis products (p = .03) [13].

In 2020, Aviram et. al did Israeli cross-sectional studies that suggested medical cannabis reduces migraine frequency over an average of three years, a self-report survey of 145 migraine patients [5]. Patients were considered responders in the trial if their mean monthly migraine days decreased by at least 50% [5]. In comparison to non-responders, respondents (n = 89, 61%) reported less current migraine disability and negative impact, as well as lower rates of opioid and triptan intake [5]. Between responders and non-responders, no statistically significant variations in demographics or migraine symptoms were found, indicating a similar baseline disease burden [5].

In terms of analgesic use and migraine disability, respondents did significantly differ [5]. Through recognized tests such as the Headache Impact Test six (HIT-6), Pittsburg Sleep Quality Index (PSQI), and Migraine Disability Scale (MIDAS), respondents demonstrated significantly less migraine disability [5]. Additionally, respondents used fewer opioids and triptans [5]. The study provides evidence that using cannabis as a migraine treatment considerably lowers the disease burden when compared to both the improvement in disability and anti-migraine drug use [5].

At the American Academy of Neurology's (AAN) Annual Meeting in 2019, a retrospective chart analysis from New York was presented, adding more evidence in favor of medical cannabis lowering migraine frequency [14]. This study of 316 patients (≥21 years of age) charts focused specifically on those who had been identified as having chronic migraines, per ICHD-3 [14]. A total of 88.3% (279) of migraine patients with an average medical cannabis exposure of 22.4±17.5 weeks said that using medical cannabis had improved their condition [14]. The average monthly migraine frequency with cannabis was reduced by 42.1% in this analysis of chronic migraine charts [14]. More than 50% of the patients reported a decrease in headache frequency, with 55.0% reporting a reduction of ≥50% in headache days [14]. Anxiety, sleep, and mood improvements were reported by patients at rates of 30.7% (97), 38.3% (121), and 24.7% (78), respectively [14].

The study by Okusanya et al. in 2022 included a total of 12 publications involving 1,980 participants from Italy and the United States of America [15]. In their studies, they concluded that medical cannabis decreased the number of days with a migraine after 30 days as well as the monthly frequency of migraine headaches [15]. Medical cannabis was 51% more effective than non-cannabis drugs for reducing migraines [15]. When compared to amitriptyline, medical cannabis reduced the frequency of migraine attacks and prevented migraine headaches in certain users (11.6%) [15]. After six months of therapy, medical cannabis also considerably decreased nausea and vomiting brought on by migraine attacks [15].

Medical Marijuana Dosing for Migraine

The recommended dosage for medicinal cannabis use varies, even though it comes in different forms.

Baron et al. in 2018 revealed a pattern of cannabis use, including frequency, quantity, and strains, in their electronic survey of the use of medical cannabis in headache patients. In the ID Migraine^TM^ questionnaire, the headache and migraine groups preferred hybrid cannabis strains, particularly 'OG Shark', which has a high THC, low CBD, and strains with a predominance of the terpenes β-caryophyllene and β-myrcene [16]. Patients in the study trial received 19% THC or 19% THC with 9% CBD as an intervention [16]. A total of 200 mg was found to be an effective amount for reducing migraine pain by 55% [16]. In a different study, patients with chronic migraines received either 25 mg of amitriptyline or 200 mg of THC and CBD daily for three months as a preventative measure; 200 mg of THC and CBD was also necessary for an acute attack [16]. The results of the study showed that 200 mg of THC with CBD was superior to amitriptyline use (40.1%) by 40.4% [16].

Mechtler et al. in 2020 studied the dosing for cannabis in migraine patients and concluded that dosing for medical cannabis is an individualized procedure that is influenced by the underlying endocannabinoid tone [1]. The suggestions moving forward are evidence-based and supported by findings from the administration of cannabis to over 13,000 patients at Dent Neurologic Institute, Buffalo, New York, a comprehensive neurologic institute [1]. Building a strong patient-physician relationship and starting cannabis therapy with a thorough understanding of the patient's etiology and treatment goals is advised [1]. Cannabis should be given in small doses at first, and then titrated up to the desired effect [1]. In a practical setting, dose titration should be carried out quickly enough to provide an effect but slowly enough not to disrupt the patient [1]. Depending on the comfort level of the patient and doctor, titration stages can be completed in one- or two-week intervals [1]. They also advised planning follow-up visits to consider any titration adjustments [1]. The option for patients to express concerns to their certifying physician in-between appointments is crucial [1]. Also, they mentioned that sublingual or inhalation cannabis (short half-life) can treat acute migraine attacks to alleviate migraine quickly, and oral formulation cannabis (long half-life) can be used for prophylactic chronic migraine treatment [1]. Doses for oral formulation cannabis should be more than those for sublingual and inhaled preparations because of their first-pass hepatic metabolism in an oral formulation [1]. For inhaled formulation, clinical considerations such as pulmonary conditions must be taken into account [1]. Sublingual dosing can be used as an alternative for such patients [1].

Finding the appropriate dosage of medical marijuana medication may be challenging due to the variations in strains, dosages, and formulations; hence, marijuana consumption should be accurately documented. Patients should be encouraged to start with a low dose, carefully monitor their response, and gradually titrate, if necessary. Inhaled or sublingual cannabis concentrate may be effective for acute migraine treatment, and oral formulation cannabis for prophylactic chronic migraine treatment.

Safety of Marijuana

Rhyne et al. conducted a retrospective chart review of 121 patients utilizing medical cannabis for migraine disease in 2016 [12]. An adverse event like somnolence was seen with an edible form of medical cannabis, which may be because of greater production of the active metabolite 11-hydroxy-tetrahydrocannabinol via hepatic metabolism of THC [12]. However, the use of medical cannabis as a migraine medication was well-tolerated and had notable impacts on frequency [12].

Mechtler et al. 2019 conducted a study of 316 patients with chronic migraine who had used medical cannabis as a treatment [14]. A total of 23.1% (73) of these cases reported adverse effects, and 1.3% (4) of those cases discontinued medical cannabis due to adverse effects. But no severe adverse effects were reported in the studies [14].

Mechtler et al. in 2022 studied the safety profile of marijuana in migraine patients [1]. Although some side effects were reported, they were mostly minor [1]. Drowsiness, lightheadedness, cognitive deficits, stomach issues, euphoria, psychosis, and headache exacerbation are among the side effects of marijuana that have been documented in his studies [1]. These represent the overdosage of cannabinoids, particularly THC. Cannabis is often not advised during pregnancy, breastfeeding, or in children [1].

In the study by Okunsanya et al. in 2022, they found mainly mild side effects in 43.75% of patients who used oral cannabinoid preparation [15]. Additionally, they reported that a large rise in dose over time for each cannabis usage session indicates medical cannabis tolerance and might lead to the use of a greater dosage of medical cannabis [15]. Medical cannabis for migraines has also been linked to medication overuse headaches (MOH) compared with non-cannabis users (81% vs 41%) [15].

A summary of the characteristics of all included studies in this review is shown in Table 2 to demonstrate the aim and findings of each article.

**Table 2:** A tabulated summary of the characteristics of included studies. USA: United States of America; UK: United Kingdom; P: probability value; MC: medical cannabis

Author	Country/Year	Study Design	Patient Character	Number of Treated Patient	Outcome
Rhyne DN et al. [12]	USA/2016	Retrospective chart review	Adult patient with migraine	121	Patients using medical cannabis for migraine disease in Colorado revealed a decrease in migraine headache frequency among 103 (85.1%) patients over an average of 21.8 months.
Baron et al. [16]	USA/2018	Literature review	Migraine patient identified after Id Migraine questionnaire	445	Among the primary illness categories, headache was the most common symptom being treated with medical cannabis. Out of 505 headache patients, 343 were identified as migraine patients after using Id migraine questionnaire. The study demonstrated the significant benefit of medical marijuana in reducing migraine-related nausea and vomiting and also may help migraine sufferers use less prescription drugs, most commonly Opioids.
Mechtler L et al. [14]	USA/2019	Retrospective chart review	Adult patient with migraine	316	279 patients reported improvement in their headache profile. Medical cannabis plays a safe role in Chronic Migraine management by helping to improve headache profile, anxiety, sleep, mood, and opioid reduction.
Aviram J et al. [5]	Israel/2020	Cross-sectional study	Adult patient with migraine	89	Medical marijuana results in long-term reduction of migraine frequency in >60% of treated patients and is associated with less disability and lower anti-migraine medication.
Stith SS et al. [10]	USA/2020	Observational study	Adult patient with migraine and headache	582 Migraine patients and 1328 headache patients	94% of users experienced symptom relief within 2 hours of observation window. Male had greater relief than female (P<0.001). Younger users (<35 years) experience greater relief than older users (P=0.08). Cannabis strain variations, user gender, headache/migraine severity, and age all had an effect on relief trends.
Cuttler et al. [8]	USA/2020	Review of Archival data on Strainprint application (App)	Adult patient with migraine	653	88.1% of patients reported a reduction in migraine severity with inhaled cannabis over the course of 16 months, as evidenced by 7441. Four hours of sessions between 653 patients. A higher pre-inhalation severity rating corresponded to greater reductions in the sample's mean migraine severity rating, which decreased by 49.6% (pre-6.65, post-3.30).
Mechtler et al. [1]	USA/2021	Literature review	Adult patient with migraine	-	Patients who used medical cannabis for migraine reported improvement in their migraine characteristics and common comorbidities. There was also a reduction in prescription medication use especially Opioids.
Gibson LP et al. [13]	USA/2022	Cross-sectional study (online survey)	Adult patient with migraine	123	Compared to non-cannabis product users, cannabis users reported significantly greater relief from migraines.
Okusanya BO et al. [15]	UK/2022	Systematic review	Adult patient with migraine	1980	Medical Cannabis (MC) was 51% more effective in reducing migraines than non-cannabis products. MC reduced the number of days of migraine after 30 days, and the frequency of migraine headaches per month. MC significantly reduced nausea and vomiting associated with migraine attacks after 6 months of use.

Limitation

There are certain limitations in our study, as we only used articles in the English language, conducted only on adult humans, and published in the last six years. Some studies had a small sample size of 89. These findings, however, may offer early insight and a framework for planning the optimization of marijuana strains, dosing, and usage pattern that may be clinically useful in the treatment of migraine.

## Conclusions

The main objective of this article is to assess the efficacy and safety of medical marijuana for the treatment of migraine headaches. All the studies showed encouraging findings on the therapeutic effects of medicinal marijuana in migraine treatment. Additionally, medical marijuana is well-tolerated with fewer side effects and is safe to use in migraine patients. More studies about the doses, frequency, and route of medical marijuana and also the inclusion of other population age groups like an adolescent and the elderly will be beneficial and worth exploring. Clinical trials with long-term follow-up are required to study more about the efficacy and safety profile of marijuana.
